# Resilient Digital Twins: Organizations Need to Prepare for the Unexpected

**DOI:** 10.1007/s12599-021-00721-z

**Published:** 2021-09-22

**Authors:** Wil M. P. van der Aalst, Oliver Hinz, Christof Weinhardt

**Affiliations:** 1 Lehrstuhl Für Informatik 9, RWTH Aachen, Ahornstr. 55, 52056 Aachen, Germany; 2 Faculty of Economics and Business Administration, Goethe University Frankfurt, Theodor-W.-Adorno-Platz 4, 60323 Frankfurt am Main, Germany; 3 Institute of Information Systems and Marketing (IISM), Karlsruhe Institute of Technology (KIT), Kaiserstr. 89-93, 76133 Karlsruhe, Germany

## Learning from Data in Times of Disruption

Currently, we can witness contradictory expectations when it comes to IT. On the one hand, there is the belief that Artificial Intelligence (AI) and Machine Learning (ML) will solve most problems because of the abundance of data and sophisticated algorithms (LeCun et al. 2015). In this editorial, we use AI/ML to refer to *machine intelligence*, i.e., mixtures of Artificial Intelligence and Machine Learning. AI/ML can deal amazingly well with unstructured data (text, images, and video) as long as there are enough training data. On the other hand, the COVID-19 pandemic and rapid climate changes (e.g., the floods in Germany in July 2021) show that AI/ML cannot deal with disruptions. When there is a sudden dramatic change, predictive models will fail, no matter how much data was there before. Consider, for example, the impact of the COVID-19 pandemic on supply chains. Especially at the beginning of the global outbreak of COVID-19 in March 2020, supply chains failed because of the unpredicted demand for certain products (e.g., masks and toilet paper) combined with simultaneous restrictions for travel, work, and business.

In times of disruption, the "training data" are not representative of the actual behavior of people and organizations. Although current AI/ML technologies outperform humans in many areas, tasks requiring common sense, contextual knowledge, creativity, adaptivity, and empathy are still best performed by humans (van der Aalst 2021b). In times of disruption, these aspects are essential. Data-driven algorithms have difficulties dealing with contextual information and data-points that are off-the-scale. Techniques for reinforcement learning can adapt automatically, but are too slow to respond to disruptions. Techniques for "transfer learning" only work if there are sufficient similarities. Hence, disruptions like COVID-19 and large-scale flooding show the limitations of machine intelligence.

Does this mean that one should simply ignore data and models because they fail when things get tough? The answer is NO for two reasons. The first reason is that if there are enough data, and processes are in steady-state, AI/ML technologies tend to function well and can take over many duties from humans. The second reason is that also in times of disruption, humans still need data and models to make good decisions. During the COVID-19 crisis, the importance of having reliable data became evident. Data were often incomplete and unreliable, complicating decision-making at all levels (Sáez et al. 2021). The General Data Protection Regulation (GDPR) further delayed actions in the European Union (Karnitschnig 2021). Also, bureaucratic processes that relied on paper forms failed. Actually, COVID-19 helped to drive digital transformation by exposing pre-existing problems. What would normally take years, now only took weeks. Interestingly, also relatively simple models helped decision-makers (e.g., high-level statistical models to predict the incidence of the coronavirus in the coming weeks).

So we would like to exploit data and be robust when confronted with an unprecedented situation. One idea would be to create a *digital twin* based on historical and current data. However, to ensure robustness, we need to add human-in-the-loop capabilities that override AI/ML algorithms when needed. A digital twin is a virtual representation that serves as the real-time digital counterpart of something that exists in the non-virtual (i.e., physical) world (e.g., a production system, machine, or organization). The digital counterpart should help to make decisions either manually or fully automatically. However, the digital twin should be *resilient* and not start making bad decisions when confronted with completely new situations. Hence, human intelligence needs to be part of such a *resilient digital twin*. Answers to "what-if analyses" need to be compared and interpreted.

## Grand Challenge: Digital Twin of an Organization (DTO)

**Fig. 1:**
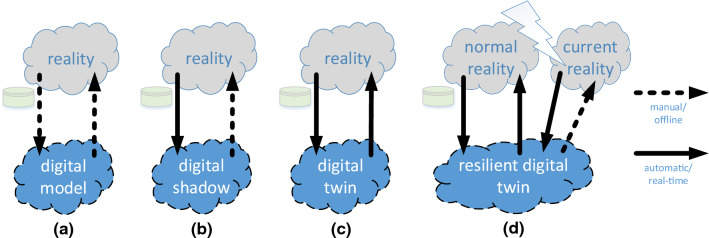
The difference between a digital model **a**, a digital shadow **b**, a digital twin **c**, and a resilient digital twin confronted with a disruption **d**. Visualization is based on (van der Aalst 2021a)

Digital twins have been used in many settings ranging from spacecraft and wind turbine simulations to chemical processes and urban planning. This editorial focuses on the *digital twin of an organization* (Kerremans and Kopcho 2019). However, we first provide a more abstract definition of a digital twin using Fig. 1 (van der Aalst 2021a; Fuller et al. 2020).

A *digital model* is a reflection of reality that is created manually and functions in an offline manner, i.e., the model does not change when reality changes. An example is the classical use of simulation tools like Arena, AnyLogic, Vensim, or Simul8 modeling a production line or supply chain. Such a digital model can be used to reason about reality and answer what-if questions. A *digital shadow* goes one step further. The model is now automatically derived and changes when reality changes. The digital shadow can also be used to reason about reality and answer what-if questions. Although the digital shadow is based on data tapped from reality, there is no automated real-time feedback loop. The insights produced by the digital shadow do not automatically trigger changes. This is still done manually after interpreting the results. The *Internet of Production* (IoP) developed at RWTH Aachen University provides collections of digital shadows supported by an infrastructure that is using AI/ML techniques with a focus on process mining (van der Aalst et al. 2021; Liebenberg and Jarke 2020). A *digital twin* goes one step further. Results of the digital twin directly impact reality. For example, when the simulation model predicts a delay, the production process is reconfigured automatically.

The idea of a digital twin is appealing. In the *virtual* world, all possible decisions can be evaluated *without* causing harm, waste, and costs. Typically, stochastic models are used to cope with uncertainty. This explains why simulation tools play a crucial role in the development of digital twins. In selected application domains, it is already possible to create reliable digital twins that can automatically respond to behaviors observed in reality. Early examples of digital twins were relatively simple, focusing on a single physical thing (e.g., a wing of an airplane). However, over time more complicated settings were considered. As discussed in recent literature, digital twins now play a key role in cyber-physical systems, Industry 4.0, Internet-of-Things, smart cities, aviation, energy, and healthcare (Fuller et al. 2020; Kritzinger et al. 2018; Caporuscio 2020). These successes led to the idea of a *Digital Twin of an Organization* (DTO).

Although the term "Digital Twin of an Organization" (DTO) existed before, it only became a topic of discussion when Gartner started to promote the concept a few years ago (Kerremans and Kopcho 2019). Gartner uses the following definition: "A digital twin of an organization (DTO) is a dynamic software model of any organization that relies on operational and/or other data to understand how an organization operationalizes its business model, connects with its current state, responds to changes, deploys resources and delivers exceptional customer value." Creating a *DTO can be seen as one of the grand challenges* in information systems.

Why is it so challenging to create a DTO? There are two main reasons:

- The boundaries of an organization and, therefore, also a DTO are not so clear, i.e., an organization has customers, suppliers, employees that collectively influence the processes.
- Human and organizational behavior may be irrational and change over time (influenced by regulations, social interactions, and personal preferences).

For most organizations, it is not feasible to create a DTO that captures reality well. However, the desire to model, visualize and understand the complex context in which an organization operates is compelling. One can view *process mining* as a concrete technology to facilitate such a DTO (van der Aalst 2016). Using process discovery, one can discover the so-called "control-flow model" (represented using Petri nets, process trees, or BPMN models) and by aligning event data with the control-flow model, it is possible to add other perspectives (time, costs, resources, decisions, etc.). The resulting more elaborate model can be simulated (van der Aalst 2016). Several process-mining tools provide such a simulation facility (e.g., ProM, Celonis, and Apromore). Also, business process modeling and simulation tools (e.g., Signavio, Aris, and Simul8) have added process-mining capabilities to automatically learn simulation models. Using process mining, it is relatively easy to create a digital shadow (see Fig. 1b). However, due to the challenges mentioned before, it is extremely difficult to create a model that behaves like a real organization. Also, multiple processes interact and compete for resources concurrently. The importance of concurrency and complex interactions between objects (customers, orders, products, machines, people, etc.) is elaborated on in Van der Aalst (2021a). Hence, it is not sufficient to consider one process in isolation. Moreover, to create a digital twin, as shown in Fig. 1c, the DTO also needs to automatically take action. *Action-oriented process mining* provides initial steps for this. For example, the Celonis Execution Management System (EMS) can trigger corrective workflows using the Integromat integration platform.

Although process mining provides initial capabilities to create a DTO, it is fair to say that, currently, DTOs are more a vision than a reality. Moreover, to make DTOs resilient, we need humans in the loop (Abdel-Karim et al. 2020) to cope with disruptions. This is illustrated in Fig. 1d. As reality changes due to disruptions, the digital twin should still be useful for human decision-makers. In other words, we want the *combination* to be resilient.

## Resilience Using Hybrid Intelligence

In Losier et al. (2019), infrastructure digital twins are proposed to cope with natural disasters such as floods, storms, fires, and earthquakes. Strictly speaking, using the classification in Fig. 1, the digital twins in Losier et al. (2019) are actually just digital models. Decision-makers can play out different scenarios to see how to respond to different types of natural disasters. This cannot be handled by AI/ML because every disaster is unique, and there are not enough representative training data. Therefore, we advocate the use of *Hybrid Intelligence* (van der Aalst 2021b).

*Hybrid Intelligence (HI)*, sometimes also called *Augmented Intelligence*, emphasizes the assistive role of AI/ML. For example, deep neural nets can be used to enhance human intelligence rather than replace it. Dellermann et al. (2019) define HI as “the ability to achieve complex goals by combining human and artificial intelligence, thereby reaching superior results to those each of them could have accomplished separately, and continuously improve by learning from each other”.

**Fig. 2:**
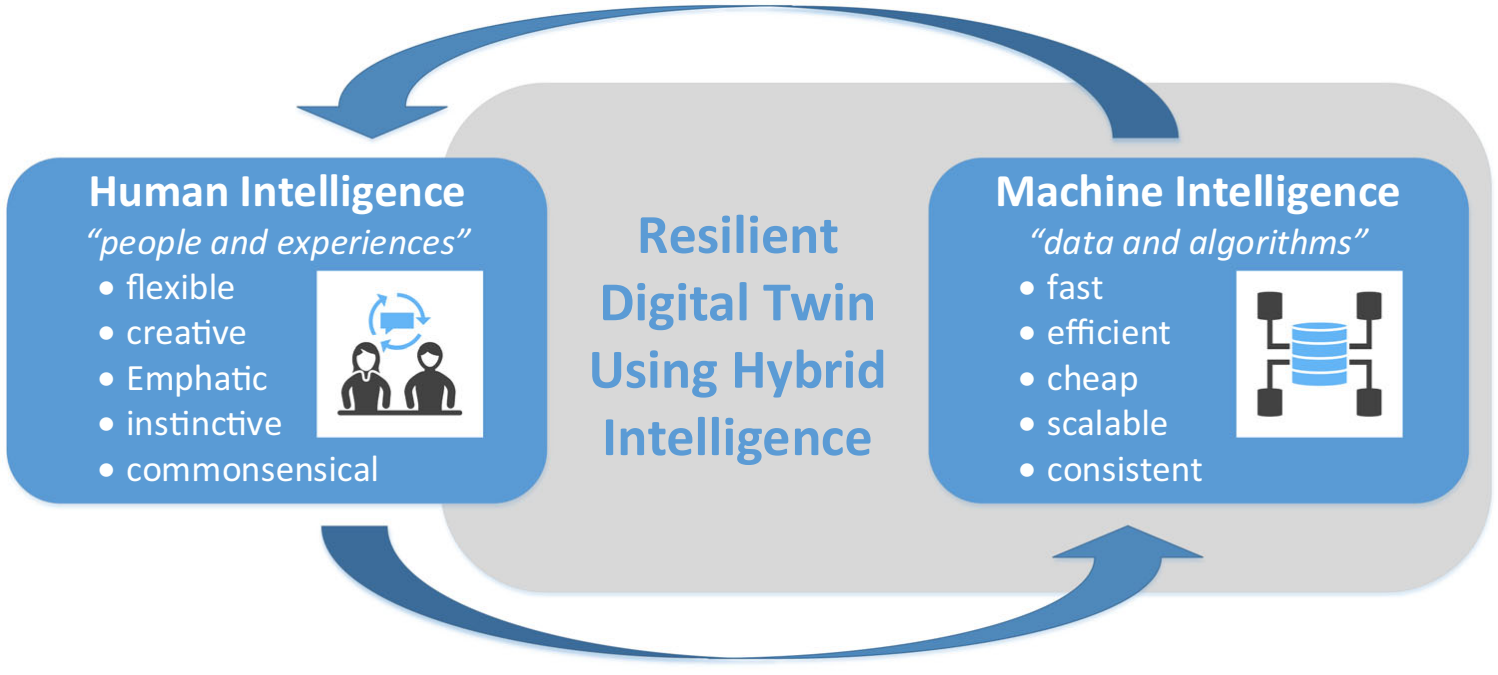
Positioning hybrid intelligence as the bridge connecting human intelligence and machine intelligence to enable resilient digital twins, based on van der Aalst (2021b)

Figure 2 shows how HI combines two forms of intelligence: *human intelligence* and *machine intelligence* (i.e., AI/ML). Human intelligence is about people and experiences and can be characterized by terms such as *flexible*, *creative*, *emphatic*, *instinctive*, and *commonsensical*. Machine intelligence is about data and algorithms and can be characterized by terms such as *fast*, *efficient*, *cheap*, *scalable*, and *consistent* (van der Aalst 2021b).

HI can help to make DTOs more resilient to disruptions. People can more easily translate past experiences into actions in unseen contexts. Instinct and common sense outperform automated reasoning when confronted with unprecedented situations.

## Relevance and Opportunities for BISE

Already in 2017, AlphaGo Zero, a Go-playing computer developed by DeepMind Technologies, was able to defeat any human player by just playing games against itself. Go is just one of many tasks where human intelligence has been outperformed by machine intelligence. Numerous studies show that many jobs will disappear in the near future because of this technological disruption (Frey and Osborne 2017; Hawksworth et al. 2018). These developments are important for the Business & Information Systems Engineering (BISE) readership. The boundaries of what is done by people and what is done by software will continue to shift. Machine intelligence will not just change the operational processes in an organization, but also the way the organization is managed and transformed. Here concepts such as Hybrid Intelligence (HI) and the Digital Twin of an Organization (DTO) will play a role. Yet, there are many open challenges for BISE researchers. Creating DTOs can be seen as one of the *grand challenges* in the field of information systems. Moreover, the COVID-19 pandemic and rapid climate changes illustrate the importance of resilience. It is not enough to create systems that are redundant and act autonomously. Human intelligence continues to play a vital role in ensuring resilience.
